# Is “end of life” a special case? Connecting Q with survey methods to measure societal support for views on the value of life‐extending treatments

**DOI:** 10.1002/hec.3640

**Published:** 2018-01-19

**Authors:** Helen Mason, Marissa Collins, Neil McHugh, Jon Godwin, Job Van Exel, Cam Donaldson, Rachel Baker

**Affiliations:** ^1^ Yunus Centre for Social Business and Health Glasgow Caledonian University Glasgow UK; ^2^ Erasmus School of Health Policy & Management Erasmus University Rotterdam Rotterdam The Netherlands; ^3^ Erasmus School of Economics Erasmus University Rotterdam Rotterdam The Netherlands

**Keywords:** end of life, Q survey, resource allocation, societal views

## Abstract

Preference elicitation studies reporting societal views on the relative value of end‐of‐life treatments have produced equivocal results. This paper presents an alternative method, combining Q methodology and survey techniques (Q2S) to determine the distribution of 3 viewpoints on the relative value of end‐of‐life treatments identified in a previous, published, phase of this work. These were Viewpoint 1, “A population perspective: value for money, no special cases”; Viewpoint 2, “Life is precious: valuing life‐extension and patient choice”; and Viewpoint 3, “Valuing wider benefits and opportunity cost: the quality of life and death.”

A Q2S survey of 4,902 respondents across the United Kingdom measured agreement with these viewpoints; 37% most agreed with Viewpoint 1, 49% with Viewpoint 2, and 9% with Viewpoint 3. Regression analysis showed associations of viewpoints with gender, level of education, religion, voting preferences, and satisfaction with the NHS.

The Q2S approach provides a promising means to investigate how in‐depth views and opinions are represented in the wider population. As demonstrated in this study, there is often more than 1 viewpoint on a topic and methods that seek to estimate that averages may not provide the best guidance for societal decision‐making.

## 1 INTRODUCTION

Legitimate arguments exist for eliciting societal values in relation to health care resource allocation given the roles of the general public as tax payers and as the potential future beneficiaries (or not) of treatments. In health economics, the main approach to eliciting societal preferences for health care has been through preference elicitation studies. Preference elicitation techniques such as willingness to pay, discrete choice experiments, or person trade‐off are based around participants making trade‐offs between options, recognising the opportunity cost of the decisions they make. However, there are challenges with the way these methods are designed. Typically, they focus on a small number of attributes and examine them ceteris paribus in hypothetically designed scenarios. This can lead to individual attributes being highlighted and potentially a higher weight given to these than if all attributes were considered together (Baker, Bateman, et al., 2010). The stylised nature of the scenarios presented in preference elicitation studies generally results in a small number of attributes, which may not capture the issues that are of importance to the members of the public who are responding to the questions. As a complement to these preference elicitation studies, there is need for a study design that can take a more holistic view on what is important to members of the public in health care decision‐making.

One topic that could benefit from this more holistic approach is around societal views on the provision of life‐extending, end‐of‐life treatments. In England, the National Institute for Health and Care Excellence (National Institute for Health and Clinical Excellence, 2009a, 2009b) introduced a policy that gave special consideration to end‐of‐life treatments. This policy was a change from the standard practice that assumes health gains measured in the form of quality adjusted life years (QALYs) are of equal value to all patients and for all disease types. With this end‐of‐life policy, QALY gains from life‐extending, end‐of‐life treatments are weighted more highly than QALY gains from other types of treatment. Evidence from decisions so far indicates a weight of approximately 1.7 that is being applied to these end‐of‐life treatments (Longson & Littlejohns, 2009).

A factor in the decision to change the policy for life‐extending, end‐of‐life treatments was the claim that society viewed end‐of‐life treatments as “different” or more valuable (Jack, 2014; Rawlins, Barnett, & Stevens, 2010). However, at the time of this decision, there was little evidence that society viewed end of life this way. Following this, a number of preference elicitation studies were conducted to gain insight into whether members of the public give additional weight to life‐extending, end‐of‐life treatments. A recent review identified 17 preference elicitation studies (12 published following the National Institute for Health and Clinical Excellence [NICE] change in policy) on this topic (Shah, 2016). Of these 17 studies, seven found a positive premium for end of life, seven found no premium, and three reported mixed findings. The mixed findings from these 17 studies may be a result of the different preference elicitation techniques, modes of administration, or differences in the questions presented to respondents, such as the size of the health gain, life expectancy, or the type of comparator presented.

However, it may also be that there is disagreement within society about the relative value of end‐of‐life treatments, which the focus on individual attributes or the aggregation of individual values in a preference elicitation study does not allow us to observe. There is a lack of in‐depth data on the *nature* of societal perspectives on the relative value of health gains at the end of life—information that may help us to explain the reasons why findings differ. Research is required that is capable of incorporating a broader range of attributes that may influence societal views. We contend that surveys underpinned by such qualitative prework, to establish “what matters” before measurement, are more likely to be robust.

In a project funded by the Medical Research Council Methodology Panel (G1002324), we conducted two interlinked studies. The objective of the first study was to identify and describe societal perspectives on the relative value of life‐extending, end‐of‐life treatments. Three viewpoints were identified and are briefly described in the next section and reported in full in McHugh et al. (2015). The objectives of the second study were to determine the distribution of these perspectives in a nationally representative sample of the U.K. population. This type of information is important if policymakers want to be responsive to public values when making decisions on developing new or reforming current guidance. This paper presents the findings of this second study. In what follows, we outline the first phase of our research, which used Q methodology to identify the three societal viewpoints of concern here. Following this, we detail how the results from the initial Q study were used to develop a survey, which was delivered online to 4,902 participants, quota sampled to reflect the U.K. population on key demographics. We present our findings with respect to distributions of the three societal viewpoints across the population. Finally, we discuss both methodological innovations and challenges and reflect on the policy implications of our results.

## 2 PRIOR Q STUDY

Q methodology combines qualitative and quantitative techniques to systematically study in‐depth, subjective views on a given subject (Watts & Stenner, 2012). As noted above, Q methodology was used to elicit and describe three viewpoints on the relative value of life extensions for people with terminal illness (McHugh et al., 2015). In brief, 59 respondents, purposively selected on the basis of their experiences, associations, or expertise, took part in a ranking exercise, ordering a set of 49 statements of opinion onto a grid. The grid comprised a corresponding number of 49 spaces presented in a pyramid or quasi‐normal distribution shape. Respondents were asked to rank the statements from “most agree” on the right hand side of the grid to “most disagree” on the left hand side. The statement set was derived from primary and secondary sources including interviews, the National Institute for Health and Clinical Excellence (2009a, 2009b) public consultation on the end‐of‐life guidance and newspaper reports related to the provision of life‐extending treatments for people with terminal illness (McHugh et al., 2015). By‐person factor analysis of the ranking data revealed three different viewpoints as described as follows, (a) “A population perspective—value for money, no special cases,” (b) “Life is precious—valuing life‐extension and patient choice,” and (c) “Valuing wider benefits and opportunity cost—the quality of life and death.”

These three viewpoints form the basis for what follows and so are described in some detail here. The first viewpoint, *A population perspective—value for money, no special cases*, is a broadly utilitarian, system‐level perspective. People associated with this point of view place importance on maximising health benefits to a population, recognising that there is a fixed health budget. According to this account, treatments yielding the greatest health improvements in relation to cost should be prioritised, as they would offer good value for money, and all patient groups must be considered equally deserving of treatment. It follows that life‐extending treatments for terminally ill patients would not usually be regarded as a cost‐effective use of resources, because health gains are typically small, and new treatments are often expensive. From this point of view, health gains for terminally ill patients are not treated as a special case and, to do so, would devalue the health of all other patients.

The second viewpoint, *Life is precious—valuing life‐extension and patient choice*, places the individual patient at the centre of decisions and is grounded in rights‐based arguments and views about entitlement. Central to this perspective is that human life is precious and that the cost of treatments should not determine provision. Patient choice should be the primary focus, and treatments must be provided if a patient chooses because everyone contributes to the funding of a public health care system. Specifically, the denial of life‐extending treatments to patients who want them is regarded as morally wrong as it does not account for patients' preferences, and the nonhealth benefits that these treatments might have for patients or their families, such as time to prepare for death, put affairs in order, or say goodbye. However, provision of end‐of‐life treatments would not be treated as a special case when making coverage decisions as the key criteria is that if a patient wants a treatment, including life‐extending treatments at the end of life, they should have it.

The third viewpoint, *Valuing wider benefits and opportunity cost—the quality of life and death*, like the first, places great emphasis on achieving best value for money with respect to limited resources, and so withholding cost‐ineffective treatments is consistent with this account. However, in this viewpoint, there is an appreciation that there may be value for patients and their families from other nonhealth benefits not captured in standard cost per QALY calculations. As such, there is room in this third account for special cases. The value attached to nonhealth benefits could mean that improvements in quality of life or life extension at the end of life are more valuable than health gains for patients with nonterminal conditions but that all treatments at the end of life are not more valuable per se.

## 3 METHODS

### 3.1 Survey design

Building on the findings of the first study, we designed a survey to measure societal support for the three viewpoints identified. The aim of Q‐based survey design (Q2S) is to extract, from the findings of an initial Q study statements that distinguish viewpoints from one another. These statements act as “markers” for those viewpoints and are used as an indication that survey respondents are more likely to agree with a particular viewpoint. The Q2S approach has been previously attempted in a small number of studies in the field of health economics (Baker, Wildman, Mason, & Donaldson, 2014; Mason, Van Exel, Baker, Brouwer, & Donaldson, 2016) but remains methodologically developmental (Baker, Van Exel, Mason, & Stricklin, 2010). To select statements that best signal agreement with the original viewpoints, there are two important considerations: salience and distinction. Salient statements are those which respondents who were highly associated with the viewpoint cared strongly about (i.e., ranked most agree or most disagree). Distinguishing statements are those which set each viewpoint apart (i.e., statements that are ranked statistically significantly differently for one viewpoint compared to another). Statements selected for the survey can be positively or negatively associated with a viewpoint but should be associated with only one and not the others. For example, considering agreement (or not) with the statements that distinguish Viewpoint 1 would indicate probable agreement with that “whole” viewpoint. The six statements do not summarise the whole viewpoint and should not be interpreted as a summary of the full ranking of all 49 statements from the original Q study. These six statements are used to signal likely agreement with that view. Applying this method, we identified 18 statements (six for each viewpoint) from the original Q study to include in the survey. Table 1 lists the 18 statements together with their position in each viewpoint in the original analysis to show how they differ for each viewpoint.

**Table 1:** Statements selected for survey (by viewpoint)

Statement number (from initial Q study)		Placement of statements for each viewpoint in the original analysis (−5 to +5 scale)		
		V1	V2	V3
	**Viewpoint 1**			
3.	Treatments should be directed towards people who have a greater chance of survival.	3	−1	0
5.	At the end of their life, patients should be cared for at home with a better quality of life rather than have aggressive and expensive treatments that will only extend life for a short period of time.	4	0	1
26.	It is wrong to raise hopes and expectations by making a special case for treatments that will only extend life by a short time.	3	−2	0
38.	The health system should be about getting the greatest benefit overall for the population.	5	0	2
2.	We should support an individual patient's choice for treatments that give short life extensions.	−3	3	−1
13.	I would place more value on end‐of‐life treatments than many medical treatments for nonterminal conditions.	−4	−2	−2
	**Viewpoint 2**			
17.	If a life‐extending treatment for terminally ill patients is expensive, but the only treatment available, it should still be provided.	−3	3	−3
20.	We all have the right to life.	1	4	−1
27.	To extend life in a way that is beneficial to the patient is morally the right thing to do.	−1	3	0
37.	All human life is precious.	1	5	0
1.	It is not worthwhile devoting more and more NHS money to someone who is going to die soon anyway.	0	−5	2
33.	End‐of‐life drugs are not a cure, they are life‐prolonging. There is no point in delaying the inevitable for a short time.	1	−4	−2
	**Viewpoint 3**			
25.	We should spend proportionately more on patients when we feel those patients have not had their fair innings—in terms of the length of their life or the quality of that life.	−3	−1	2
31.	Treatments that are very costly in relation to their health benefits should be withheld.	1	−5	3
34.	Patients at the end of life will grasp any slightest hope but that is not a good reason for the NHS to provide costly treatments that may extend life by a short time.	2	−3	4
41.	I would not want my life to be extended just for the sake of it—just keeping breathing is not life.	3	0	5
23.	A year of life is of equal value for everyone.	0	0	−4
24.	You cannot put a price on life.	0	3	−3

*Note*. Grey shaded statements are negatively associated with the viewpoint.

The survey was delivered online. To introduce the survey, a short animation was created. 1The short animation can be found here: http://www.gcu.ac.uk/endoflife/onlinesurvey/introductoryanimation/. This explained the premise of the survey to respondents and the context of there being limited resources for health care and a need to make decisions about which treatments and services will be provided. It went on to explain that there are many different things that could be considered when making decisions about how best to allocate resources, such as severity of illness or quality of life, and that in this research, the focus was on treatments that help terminally ill patients live longer.

Following the video, the 18 statements were presented, three per screen (one per factor) in random order, and respondents were instructed to indicate their agreement with each statement on a Likert scale labelled *completely disagree* (1) to *completely agree* (7). 2The survey is accessible at http://www.gcu.ac.uk/endoflife/onlinesurvey/q2ssurveyapproach1/. The results reported here are from one section of a longer questionnaire in which different approaches to Q2S survey design and policy choice questions were devised.

Following the completion of the survey, respondents were presented with 15 further general questions. These included standard demographics such as age, gender, and income along with characteristics that could plausibly relate to respondents' viewpoints, including voting preferences, religion, satisfaction with the NHS, and experience with terminal illness as of May 2014 (the full list is presented in Table A1).

To test the validity of our survey instrument, a subsample of 134 people was recontacted and asked to complete a full Q sort. The purpose of this external validity test was to check directly if the viewpoint that we aligned a respondent to using the Q2S survey was the same viewpoint that they would have been aligned with if they ranked all 49 statements in the Q sort. Results for both approaches were computed and correlations between the two compared. The results of the validity test are presented in Section 4.

### 3.2 Data collection

The online survey was programmed and delivered by YouGov (http://www.yougov.co.uk) in May 2014. Respondents were quota sampled from their online panel to reflect the make‐up of the U.K. population 3We recognise that online respondent panels cannot be said to be representative of the wider population (in the same way that survey respondents, focus group participants, and all methods are subject to degrees of self‐selection bias) but aimed nonetheless, to represent key characteristics in proportion to the general population. in terms of age, gender, ethnicity, and socio‐economic status.

### 3.3 Analysis

In an attempt to exclude respondents who may not have fully engaged with the online survey, those who completed the whole questionnaire very quickly (less than 7 min and 30 s) or very slowly (longer than 2 hr) were excluded from the analysis. Although these cut‐offs are arbitrary, and it is possible that we excluded valid and considered responses, the research team tested and timed the survey and judged those completing faster than 7 min and 30 s as unlikely to have read and processed all information presented. Likewise, we made a judgement that those who spent more than 2 hr on the survey may not have been fully engaged and may not fully recall the aim of the survey outlined in the animated introduction when they first started.

Following the collection of the data, Cronbach's α was calculated to test the internal consistency of the six statements representing each viewpoint. This analysis indicated that Statement 25 (see Table 1) did not share the same underlying construct as the other five statements selected to represent Viewpoint 3. Therefore, scores for Statement 25 were not used, and the remaining five statements were used in the analysis. For Viewpoints 1 and 2, all six statements were retained for the analysis. Scores for the “negative” statements were inverted before further analysis was conducted, so that all scores were on the same scale.

Each individual's Likert scale scores (between 1 and 7) were summed for the block of statements related to each viewpoint. This gave each respondent a score between 6 and 42 for Viewpoints 1 and 2 and between 5 and 35 for Viewpoint 3. To account for the differential number of statements representing Viewpoint 3, the total scores per viewpoint were rescaled linearly between 0 and 10. Finally, respondents were “assigned” to one of the original three viewpoints based on which viewpoint resulted in the highest score. Respondents who scored less than 4 (out of 10) on all viewpoints were excluded from further analysis. To gain a total score under 4, respondents would have had to rate the majority of the statements in the “disagree” end of the Likert scales, indicating that they could not find their views represented in any of the statements. Therefore, it seemed inappropriate to assign them to one of the three viewpoints and were thus removed from further analysis.

Multinomial logistic regression (MNL) was used to examine the relationship between respondents' viewpoints, as assigned above, and their sociodemographic characteristics and responses to the follow‐up questions as outlined in Table A1. MNL was used for the analysis because the dependent variable can take more than two, not logically ordered, discrete values, in this case Viewpoints 1, 2, or 3. Viewpoint 1 was chosen as the comparator (against which others are compared) because it best represents current NICE decision‐making for all other non‐end‐of‐life technologies, and gaining insight into the particular characteristics or experiences of those not associated with this viewpoint would aid our understanding of why they hold a divergent point of view from current policy. Those with an equal highest score on more than one viewpoint (categorised as “mixed”) were excluded from the MNL regression analysis due to the small numbers. The variables listed in Table A1 were included in the regression analysis, but for concise presentation, only the variables that were significant at the 10% level are included in Table 4. The relative risk in this case indicates the change in likelihood of being associated with a viewpoint, for a one unit increase in the independent variable when all other variables remain constant.

### 3.4 Sensitivity analysis with exemplars

Respondents were generally assigned to a single viewpoint based on the viewpoint associated with their highest total Likert score (once adjusted for the number of statements as previously outlined). The decision to allocate respondents to a single viewpoint based on highest score was a pragmatic one, to allow us to examine the prevalence of the viewpoints within the sample. However, respondents have a score for all three viewpoints, and in some cases, these scores may be very close. With that in mind, we identified a subsample of respondents who might be regarded as “exemplars” for a viewpoint. This is a concept drawn from Q methodology, where exemplars are respondents who most “purely” associate with a viewpoint.

To select exemplars, we calculated the average score for each viewpoint across the whole sample. Exemplars were then defined as those with a score greater than the mean for one viewpoint, and scores on the other two viewpoints were less than their highest score by 1.5 points (on the 0–10 scale). For example, the mean rescaled score for Viewpoint 1 was 6.0. To be defined as an exemplar of Viewpoint 1, a respondent had to have a maximum score for Viewpoint 1 greater than or equal to 6 and scores for both Viewpoints 2 and 3 at least 1.5 points lower than the score for Viewpoint 1.

As a form of sensitivity analysis, we examined the relationship between respondent characteristics and viewpoints based on this “exemplar subsample” only. We compared these findings with those from the whole—“highest score” rule—sample, to test whether the associations of viewpoints with characteristics are robust.

### 3.5 Ethics

Ethical approval for this study was obtained from the School of Health and Life Sciences Ethics Committee, Glasgow Caledonian University (Reference B11/04).

## 4 RESULTS

### 4.1 Sample

The total number who completed the survey was 5,496. The application of the exclusion criteria based on time to complete the survey excluded 566 respondents; 21 incomplete respondents were also excluded; and 7 scoring less than 4 on all statements were excluded. These exclusions resulted in a final sample of 4,902, which is presented in Table 2 by sampling variables. A full description of the sample, by response to each of the 15 questions included at the end of the survey, is given in Table A1.

**Table 2:** Sociodemographic characteristics of respondents by matched viewpoints

Variables	Total sample		Viewpoint								
			1		2		3		Mixed		*p*
	*N*	%	*N*	%^	*N*	%	*N*	%	*N*	%	
	**4,902**		1,806	36.8	2,416	49.3	449	9.2	231	4.7	
Age											<.001**
18**–**29	**761**	**15.5**	205	11.4	421	17.4	81	18.0	54	23.4	
30–49	**1,642**	**33.5**	517	28.6	907	37.5	133	29.6	85	36.8	
50–64	**1,304**	**26.6**	520	28.8	602	24.9	138	30.7	44	19.0	
65–74	**678**	**13.8**	309	17.1	296	12.3	48	10.7	25	10.8	
75+	**517**	**10.5**	255	14.1	190	7.9	49	10.9	23	10.0	
Gender											<.001**
Male	**2,438**	**49.7**	963	53.3	1,096	45.4	254	56.6	125	54.1	
Female	**2,464**	**50.3**	843	46.7	1,320	54.6	195	43.4	106	45.9	
Education											<.001**
Low education	**1,263**	**25.8**	472	26.1	649	26.9	76	16.9	66	28.6	
Middle education	**1,257**	**25.6**	451	25.0	613	25.4	134	29.8	59	25.5	
High education	**2,253**	**46.0**	847	46.9	1,082	44.8	232	51.7	92	39.8	
Income											.109
Low income	**1,222**	**24.9**	452	25.0	605	25.0	95	21.2	69	29.9	
Middle income	**1,405**	**28.7**	534	29.6	686	28.4	120	26.7	65	28.1	
High income	**1,067**	**21.8**	407	22.5	509	21.1	118	26.3	33	14.3	
Ethnicity											<.001**
White	**4,437**	**90.5**	1,682	93.1	2,147	88.9	409	91.1	199	86.1	
Non‐White	**365**	**7.4**	101	5.6	212	8.8	27	6.0	25.0	10.8	
Country											.094*
England	**4,065**	**82.9**	1,471	81.5	2,011	83.2	386	86.0	197	85.3	
Wales	**243**	**5.0**	103	5.7	117	4.8	12	2.7	11	4.8	
Scotland	**513**	**10.5**	207	11.5	245	10.1	44	9.8	17	7.4	
Northern Ireland	**81**	**1.7**	25	1.4	43	1.8	7	1.6	6	2.6	
Socio‐economic group (SEG)											.206
AB (SEG)	**1,464**	**30.0**	559	31.1	705	29.3	156	34.9	44	19.1	
C1 (SEG)	**1,469**	**30.1**	536	29.8	716	29.7	136	30.4	81	35.2	
C2 (SEG)	**807**	**16.5**	295	16.4	410	17.0	61	13.6	41	17.8	
DE (SEG)	**1,144**	**23.4**	410	22.8	576	23.9	94	27.8	64	27.8	

*Note*. NB: For some questions there was an option to answer “do not know” or “prefer not to say,” these responses are not included in the table and may affect the numbers adding up to the total sample.

** 1% significance level.

* 10% significance level.

^ %'s are those within viewpoint, for example, 53.3% of those aligned with Viewpoint 1 are male.

The bold type indicated the results of the full sample. The non‐bold type are the results by Viewpoint.

### 4.2 External validity testing

The correlation between the survey scores on each viewpoint and the full Q sort was .74. If we took the results of the full Q sort as being the respondents' “true” viewpoint, the survey correctly predicted which viewpoint a respondent was aligned with 74% of the time. This seems to indicate a good overall level of alignment between the survey instrument and the full Q sorts.

### 4.3 Summary statistics

**Figure 1:**
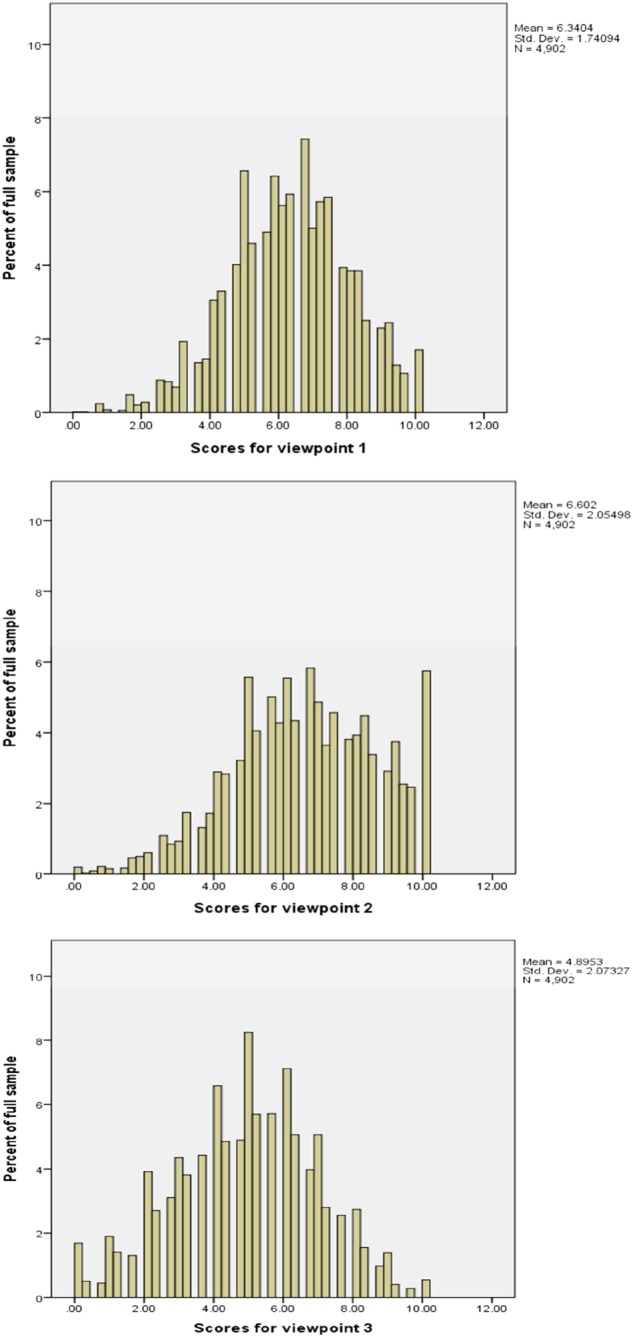
Distribution of scores, by viewpoint [Colour figure can be viewed at http://wileyonlinelibrary.com]

Descriptive statistics for each viewpoint are presented in Table 3. Paired sample tests compare the means for each viewpoint (1 and 2, 2 and 3, and 1 and 3). This test shows that all means were statistically significantly different (*p* < .001) from zero. Figure 1 shows the distribution of scores for each viewpoint. For all viewpoints, the distribution was left skewed, but this pattern can be seen more clearly for Viewpoint 2 where approximately 6% of those associated with this viewpoint scored 10 compared with less than 2% and less than 1% for Viewpoints 1 and 3, respectively. Descriptive statistics for each statement are presented in Table A2.

**Table 3:** Summary of rescaled Likert scores by viewpoint

	Descriptive statistics							Correlations	
	*N*	Range	Mean	*SD*	Med	Skewness	Kurtosis	Viewpoint 2	Viewpoint 3
Viewpoint 1	4,902	0–10	6.34	1.74	6.39	−0.197	−0.117	−.676	.698
Viewpoint 2	4,902	0–10	6.60	2.06	6.67	−0.284	−0.361		−.776
Viewpoint 3	4,902	0–10	4.89	2.07	5.00	−0.122	−0.363		

*Note*. *SD* = standard deviation.

### 4.4 Prevalence of viewpoints

The majority of respondents (95.3%) had a maximum score on either Viewpoints 1, 2, or 3: 36.8% (*n* = 1,806) of respondents were matched with Viewpoint 1 (*A population perspective—value for money, no special cases*); 49.3% (*n* = 2,416) were matched with Viewpoint 2 (*Life is precious—valuing life‐extension and patient choice*); and 9.2% (*n* = 456) were matched with Viewpoint 3 (*Valuing wider benefits and opportunity cost—the quality of life and death*). The remaining respondents (4.7%, *n* = 231) had equal maximum scores (all of 5 or above) for more than one viewpoint (and were assigned to the category mixed).

### 4.5 Examining the relationship between viewpoints and other respondent characteristics

The results of the MNL regression analysis presented in Table 4 indicate that associations with different viewpoints were linked to some individual characteristics. Relative to respondents assigned to Viewpoint 1, those assigned to 2 were more likely to be female, belong to a religion, vote labour, have a lower education level and be more dissatisfied with the NHS. Respondents assigned to Viewpoint 3 were more likely to have a higher education level and be more dissatisfied with the NHS but less likely to be female and to belong to a religion.

**Table 4:** Relationship between the viewpoints and respondents' characteristics

Characteristic	Viewpoint^				
	2*N* = 2,416		3*N* = 449	
	RRR	*p*	RRR	*p*
Age (years)	0.98 (0.97–0.98)	<.001***	0.99 (0.98–1.00)	.004***
Sex (female)	1.21 (1.06–1.37)	<.001***	0.81 (0.67–1.01)	.062*
Education level (low = no qualifications to pre‐GCSE, middle = GCSE to diploma, or high = undergraduate degree and above)	0.91 (0.84–0.98)	.014**	1.18 (1.03–1.35)	.018**
Religion: Do you regard yourself as belonging to any particular religion? Yes or no.	1.53 (1.34–1.75)	<.001***	0.67 (0.53–0.84)	<.001***
Over 65 s in household (yes or no)	1.18 (0.99–1.41)	.056*	0.98 (0.73–1.32)	.889
Satisfaction with NHS—1 = very satisfied to 5 = very dissatisfied	1.13 (1.06–1.21)	<.001***	1.15 (1.03–1.28)	.013**
Conservative voter	0.67 (0.57–0.79)	<.001***	1.17 (0.91–1.50)	.232
Labour voter	1.38 (1.18–1.62)	<.001***	0.98 (0.74–1.29)	.896

*Note*. GCSE = General Certificate of Secondary Education.

*** 1% significance level.

** 5% significance level.

* 10% significance level.

^ Viewpoint 1 is the comparator.

### 4.6 Identifying exemplars

Following the methods outlined in the analysis section, 2,423 respondents were identified as exemplars, with 651 (27%) exemplar respondents for Viewpoint 1; 1,701 (70%) for Viewpoint 2; and 71 (3%) for Viewpoint 3. Using this subset of individuals in the MNL analysis, we can compare those who are most strongly and purely associated with each viewpoint and their characteristics. The results are shown in Table 5.

**Table 5:** Relationship between those strongly associated with the viewpoints and respondents characteristics

Characteristic	Viewpoint^			
	2*N* = 1,701		3*N* = 71	
	RRR	*p*	RRR	*p*
Age (years)	0.97 (0.96–0.97)	<.001***	0.99 (0.97–1.00)	.130
Sex (female)	1.25 (1.03–1.52)	.024**	0.66 (0.39–1.11)	.130
Education level (low = no qualifications to pre‐GCSE, middle = GCSE to diploma, or high = undergraduate degree and above)	0.89 (0.80–1.00)	.062*	1.13 (0.82–1.55)	.452
Religion: Do you regard yourself as belonging to any particular religion? Yes or no.	1.77 (1.44–2.17)	<.001***	0.57 (0.32–1.02)	.057*
Over 65 s in household (yes or no)	1.09 (0.84–1.40)	.521	0.60 (0.28–1.29)	.188
Satisfaction with NHS—1 = very satisfied to 5 = very dissatisfied	1.16 (1.05–1.28)	<.001***	1.14 (0.89–1.47)	.291
Conservative voter	0.62 (0.48–0.78)	<.001***	1.22 (0.67–2.24)	.520
Labour voter	1.42 (1.12–1.81)	<.001***	0.98 (0.51–1.89)	.957

*Note*. GCSE = General Certificate of Secondary Education.

*** 1% significance level.

** 5% significance level.

* 10% significance level.

^ Viewpoint 1 is the comparator.

For Viewpoint 3, belonging to a religion is now the only statistically significant variable. However, it should be noted that the number of exemplars associated with Viewpoint 3 is only 71. Generally, the results for Viewpoint 2 were very similar to the original analysis presented in Table 4; only having over 65 s in the household was no longer statistically significant.

## 5 DISCUSSION

This paper reports the findings of a survey based on a prior Q‐methodology study, investigating the distribution of societal viewpoints with respect to the provision of life‐extending treatments for people with terminal illness. We found two dominant viewpoints (1 and 2) that were preferred by 37% and 49% of the respondent sample, respectively. There was less support for Viewpoint 3 (9%). This reflects a well‐documented, ethical “divide” between a broadly utilitarian perspective that focuses on maximising health outcome in the context of a fixed budget and a perspective based on patient choice and entitlement to health care that would likely result in an increased health budget, if operationalised.

These results could be seen as presenting a dilemma for policymakers as they indicate that there is not a majority viewpoint. This is not necessarily to suggest that policy making *should* be majoritarian, or that the viewpoint that is most common amongst respondents is the only legitimate basis for policy—but, we would argue, that both the nature of social perspectives on a given policy topic and the level of agreement with those perspectives in the wider population are important pieces of information in devising policies. The identification of two well‐supported viewpoints, in itself, could be viewed as important new knowledge. There are dominant voices in the debate around the provision of life‐extending, end‐of‐life treatments that mirror Viewpoints 1 and 2. A number of claims have been made about the views of the general public, generally in support of giving additional priority to treatments for cancer or terminal illness (Jack, 2014; Rawlins et al., 2010). However, this has often been conjecture, based on the lack of protest from other patient groups, or the general public more widely, to changes in policy.

This project (including the paper by McHugh et al., 2015) was designed to identify, describe, and examine the distribution of societal values in relation to the provision of life‐extending treatments for people with terminal illnesses. The results indicate that current policies including the NICE End‐of‐Life Guidance and the Cancer Drug Fund in England would find very little support and that the viewpoints suggest what type of policies might find traction in the U.K. public. Viewpoint 2 suggests that end‐of‐life treatments should be provided if patients choose them, but it does not make end of life a special case as these policies do, instead all treatments should be available if patients wish to have them—a view that would require increases in the health budget. In Viewpoint 3, life extension for people with a terminal illness may be valued more than the health gains for patients with nonterminal illness, but the standard approaches to assessing health benefits do not fully capture these for use in the decision‐making process. Only one in 10 of our sample most agreed with Viewpoint 3, which could be considered to be more nuanced and technical, focussing on the specifics of how we measure wider benefits of health interventions. It is possible that people who have not thought about this issue before, completing a survey might tap into the broad distinctions of Viewpoints 1 and 2, and thus, Viewpoint 3 receives less general support in a larger general public sample.

Although these results may not support current policies, they do raise challenges for decision‐making. Viewpoint 2 in particular, grounded in patient choice, would necessitate growing the health care budget. In both phases of this work, the introduction to the survey stressed that the NHS has a limited budget and that decisions need to be made about how best to spend this. Despite this, Viewpoint 2 rejects the traditional health economics approaches to decision‐making that considers costs and outcomes together to provide interventions that are good value for money. Viewpoint 2 is most certainly an account that rejects cost as a deciding factor—and that is problematic (for health economists and for policymakers), but the qualitative data shone a different light on its interpretation. What appeared to be a denial of resource constraint altogether, and perhaps misunderstanding of the issues, was shown in the qualitative data (as reported in McHugh et al., 2015), to be a political argument that in a rich country we can afford more—political decisions determine the size of the budget, and other decisions are possible. On the basis of the qualitative findings, we interpret this as a relevant and not a confused account. It feels counterintuitive to us as health economists who are used to designing preference elicitation studies that do not allow for a response like Viewpoint 2. However, as the introductory sections of the paper show, there is a very mixed empirical literature on end of life. The intention in this programme of work was to try and gain insight into why this might be the case. By allowing respondents to express a view that may seem “irrational” considering the premise of the survey, we have generated new knowledge that would not emerge from standard preference elicitation studies. The statements in the Q set come from in‐depth interviews, and so it could be argued that the viewpoints revealed in this work are more “emergent” or grounded in data rather than imposed by constraints built into our study design. We take the view that, however, inconvenient Viewpoint 2, it is important to understand it. We agree that it is not operational in a policy sense within the short term, but it is surely better to know about these views so that they can at least be addressed in policy discourse.

With the increasing importance of involving the public in health care decision‐making, there is an opportunity for work like this to be used to inform selection of participants for public and patient involvement in groups such as NICE's Citizen Council. Instead of sampling in a way that aims to approximate different views via sociodemographic and other characteristics, participants could be selected to represent a range of perspectives on the issues at stake. The results of our MNL regression analysis indicated that there were only a small number of sociodemographic characteristics that influenced that the viewpoint respondents were most associated with. Thus, selection of participants for groups like the Citizen Council based on sociodemographic characteristics may not be inclusive of the full range of views that exist on issues that would guide resource allocation. Surveys such as the one presented in this paper could be used as a selection tool, along with other sampling procedures based on sociodemographics, so that there is a balance of viewpoints in the participants selected.

This project also had a methodological aim and has contributed to the development of Q2S methods. Q2S is still a relatively unused approach to eliciting societal views in large samples, with only a small number of published applications of Q‐based survey methods (Brown, 2002; Mason et al., 2016; Talbott, 1963). The use of Likert scales to score statements has been previously used in only two studies with difficulties in presenting the methods of analysis acknowledged (Baker, Bateman, et al., 2010; Baker, Van Exel, et al., 2010); therefore, a new approach to analysis was designed for this study. The approach taken to assign respondents to a viewpoint based on highest score was somewhat pragmatic, allowing us to categorise respondents in order to examine the support for each viewpoint. However, a limitation of this approach is that we lose information on respondent's strength of association with each of the viewpoints. To investigate how strength of association with each viewpoint may influence our overall results, we conducted the “exemplar analysis.” In future research, we will build on these methods and look to other fields, such as electoral voting systems, to incorporate into the design and analysis of Q2S methods the fact that individuals can be associated (to different degrees) with more than one viewpoint.

As in all empirical studies, there are a number of strengths and limitations of the methods. Large scale attitudinal or valuation surveys are often designed according to attributes thought to be of interest or based in theoretical concepts. A strength of this work is that the constructs measured in this survey emerge directly from empirical research. The three views that form the basis of this survey arose from the previous Q study, which combined qualitative and quantitative elements. This allowed us to explore in depth what views exist on the relative value of life‐extending treatments for people with terminal illness. However, the Q2S design rests on the premise that association with a whole viewpoint can be approximated by selecting key aspects of that viewpoint and using those as indicators of the whole viewpoint. This is somewhat contentious in Q methodological terms, where the emphasis is on interpretation of the viewpoints based on the placement of all of the statements. Extracting statements from that set, it could be argued, alters their meaning, and therefore, one criticism of this approach is that salient and distinguishing statements cannot be used in isolation to signal agreement with a more complex perspective. The external validity testing outlined in Section 3 indicated that there is reasonably good alignment between the viewpoints that we would expect a respondent to be aligned to based on the results of their full Q sorts and the survey. This was particularly the case for Viewpoint 2. Examining the correlations between the survey results and the full Q sort according to each viewpoint, the correlation for Viewpoint 2 was higher at .8, and the survey correctly predicted 85% of the respondents aligned with Viewpoint 2 in the Q study. For Viewpoints 1 and 3, the correlations were lower at .75 (73% correctly predicted) and .71 (44% correctly predicted), respectively. The ability to distinguish between respondents aligned with Viewpoints 1 and 3 may be lower in this survey as a result of a high correlation of .68 between Viewpoints 1 and 3 in the initial Q study. Viewpoints 1 and 3 have in common a central focus on achieving value for money. However, for Viewpoint 3, quality of life is a crucial focus, and its value is interpreted more widely, although in both accounts, technologies should not be provided if there is not significant benefit, even if that means denying patients access to certain treatments. This high correlation impacted on the survey design as it resulted in a smaller number of statements that were both distinguishing and salient for Viewpoint 3. Given the approach to scoring each viewpoint, this could potentially have led to respondents who would have been aligned with Viewpoint 3 in a Q sort being categorised as Viewpoint 1 or mixed, thus underestimating the prevalence of Viewpoint 3.

The statements that were used in both the Q study and this survey were designed to cover the range of opinions that exist on the relative value of end‐of‐life treatments, and as such, some can be quite complex. The statements were piloted as part of the Q study, but in this Q2S survey where respondents only saw a selection of statements and completed a less intensive task, it may have created less engagement and therefore less awareness of the implications of their choices.

The design of this survey used Likert scales as the method to score each statement as the data that they provide can be used flexibly and they are common in survey research. However, Likert scales do not force the participant to make trade‐offs as is the case in Q methodology studies where statements must be rank ordered. The selection of statements as markers for different viewpoints results in a group of statements that could be seen as quite contradictory; thus, if participants gave the same score to all statements, it is likely to indicate that they have not engaged with the exercise, and we removed these participants from the final analysis. Other Q‐based survey approaches that did require participants to rank statements were tested as part of this programme of work but are not presented here (these can be accessed at http://www.gcu.ac.uk/endoflife/onlinesurvey/). Participant engagement was also assessed through the use of the time taken to complete the survey. Participants who completed the survey very fast (<7.5 min) or slowly (>2 hr) were excluded from the final analysis. This is an imperfect way to assess engagement, and there may be respondents close to the thresholds who did not engage well but were included in the data set as well as engaged but slow participants who were excluded.

This approach can be viewed as a complement to traditional preference elicitation studies such as those described in Section 1. The first phase of this work highlighted that there are multiple viewpoints in relation to the relative value of life‐extending treatments, and in this study, we have demonstrated that there is not one dominant viewpoint. This could help to explain why the preference studies in this field have yielded such conflicting findings even if the results could be considered problematic for health economists and policymakers.

This study has provided the most comprehensive examination of the Q2S approach to date while generating new evidence on the views of the U.K. general public on a highly contested policy issue. The Q2S approach provides a promising means to investigate how in‐depth views and opinions (which by their nature need to be captured through smaller, qualitative studies) are represented in the wider population. This approach retains the plural nature of the views that exists on a particular topic, which is an advantage of the Q2S approach. As demonstrated in this study, there is often more than one viewpoint on a topic and methods that seek to estimate that averages may not provide the best guidance for societal decision‐making (Van Exel, Baker, Mason, Donaldson, & Brouwer, 2015). Although we have demonstrated the Q2S approach with examination of societal viewpoints on the relative value of treatments for people with a terminal illness, this method can be used to study any topic where we expect there to be differing viewpoints within society, and there is a policy interest to understand how strongly these views are supported within the wider public.

## CONFLICT OF INTEREST

The authors declare that they have no conflict of interest.

## Supporting information

Table A1: Full list of demographics and responses by viewpoint
Table A2: Descriptive statistics by statement
