# Supplementary material for: Is “end of life” a special case? Connecting Q with survey methods to measure societal support for views on the value of life‐extending treatments
Source: Health Econ. 2018 Jan 19;27(5):819–31. doi: 10.1002/hec.3640 (PMC5900899; doi:10.1002/hec.3640)
Supplement: Supplementary file 1 — Table A1: Full list of demographics and responses by viewpoint Table A2: Descriptive statistics by statement [file HEC-27-819-s001.docx]

**Supplementary Appendix**

**Table A1: Full list of demographics and responses by viewpoint**

| **Variables** | **Total Sample** | |  | **Viewpoint** | | | | | | | | |
| --- | --- | --- | --- | --- | --- | --- | --- | --- | --- | --- | --- | --- |
|  |  |  |  | **1** | | **2** | | **3** | | **Mixed** | | **P** |
|  | **N** | **%** |  | **N** | %* | **N** | % | **N** | % |  |  |  |
| **Age** |  |  |  |  |  |  |  |  |  |  |  | <0.001 |
| 18**-**29 | **761** | **15.5** |  | 205 | 11.4 | 421 | 17.4 | 81 | 18.0 | 54 | 23.4 |  |
| 30-49 | **1,642** | **33.5** |  | 517 | 28.6 | 907 | 37.5 | 133 | 29.6 | 85 | 36.8 |  |
| 50-64 | **1,304** | **26.6** |  | 520 | 28.8 | 602 | 24.9 | 138 | 30.7 | 44 | 19.0 |  |
| 65-74 | **678** | **13.8** |  | 309 | 17.1 | 296 | 12.3 | 48 | 10.7 | 25 | 10.8 |  |
| 75+ | **517** | **10.5** |  | 255 | 14.1 | 190 | 7.9 | 49 | 10.9 | 23 | 10.0 |  |
| **Gender** |  |  |  |  |  |  |  |  |  |  |  | <0.001 |
| Male | **2,438** | **49.7** |  | 963 | 53.3 | 1,096 | 45.4 | 254 | 56.6 | 125 | 54.1 |  |
| Female | **2,464** | **50.3** |  | 843 | 46.7 | 1,320 | 54.6 | 195 | 43.4 | 106 | 45.9 |  |
| **Education** |  |  |  |  |  |  |  |  |  |  |  | <0.001 |
| Low education | **1,263** | **25.8** |  | 472 | 26.1 | 649 | 26.9 | 76 | 16.9 | 66 | 28.6 |  |
| Middle education | **1,257** | **25.6** |  | 451 | 25.0 | 613 | 25.4 | 134 | 29.8 | 59 | 25.5 |  |
| High education | **2,253** | **46.0** |  | 847 | 46.9 | 1,082 | 44.8 | 232 | 51.7 | 92 | 39.8 |  |
| **Income** |  |  |  |  |  |  |  |  |  |  |  | 0.109 |
| Low income | **1,222** | **24.9** |  | 452 | 25.0 | 605 | 25.0 | 95 | 21.2 | 69 | 29.9 |  |
| Middle income | **1,405** | **28.7** |  | 534 | 29.6 | 686 | 28.4 | 120 | 26.7 | 65 | 28.1 |  |
| High income | **1,067** | **21.8** |  | 407 | 22.5 | 509 | 21.1 | 118 | 26.3 | 33 | 14.3 |  |
| **Ethnicity** |  |  |  |  |  |  |  |  |  |  |  | <0.001 |
| White | **4,437** | **90.5** |  | 1,682 | 93.1 | 2,147 | 88.9 | 409 | 91.1 | 199 | 86.1 |  |
| Non-white | **365** | **7.4** |  | 101 | 5.6 | 212 | 8.8 | 27 | 6.0 | 25.0 | 10.8 |  |
| **Country** |  |  |  |  |  |  |  |  |  |  |  | 0.094 |
| England | **4,065** | **82.9** |  | 1,471 | 81.5 | 2,011 | 83.2 | 386 | 86.0 | 197 | 85.3 |  |
| Wales | **243** | **5.0** |  | 103 | 5.7 | 117 | 4.8 | 12 | 2.7 | 11 | 4.8 |  |
| Scotland | **513** | **10.5** |  | 207 | 11.5 | 245 | 10.1 | 44 | 9.8 | 17 | 7.4 |  |
| Northern Ireland | **81** | **1.7** |  | 25 | 1.4 | 43 | 1.8 | 7 | 1.6 | 6 | 2.6 |  |
| **Socio-economic class** |  |  |  |  |  |  |  |  |  |  |  | 0.206 |
| AB (SEG) | **1,464** | **30.0** |  | 559 | 31.1 | 705 | 29.3 | 156 | 34.9 | 44 | 19.1 |  |
| C1 (SEG) | **1,469** | **30.1** |  | 536 | 29.8 | 716 | 29.7 | 136 | 30.4 | 81 | 35.2 |  |
| C2 (SEG) | **807** | **16.5** |  | 295 | 16.4 | 410 | 17.0 | 61 | 13.6 | 41 | 17.8 |  |
| DE (SEG) | **1,144** | **23.4** |  | 410 | 22.8 | 576 | 23.9 | 94 | 27.8 | 64 | 27.8 |  |
| **Religion** |  |  |  |  |  |  |  |  |  |  |  | <0.001 |
| Belong to religion - No | **2,525** | **51.5** |  | 956 | 52.9 | 1,152 | 47.7 | 292 | 65.0 | 125 | 54.1 |  |
| Belong to religion - Yes | **2,376** | **48.5** |  | 850 | 47.1 | 1,263 | 52.3 | 157 | 35.0 | 106 | 45.9 |  |
| **Actively practising religion** |  |  |  |  |  |  |  |  |  |  |  | 0.01 |
| No | **1,370** | **57.7** |  | 521 | 61.4 | 689 | 54.6 | 102 | 65.0 | 58 | 54.7 |  |
| Yes | **899** | **37.9** |  | 306 | 36.0 | 505 | 40.0 | 49 | 31.2 | 39 | 36.8 |  |
| **Household size** |  |  |  |  |  |  |  |  |  |  |  | <0.001 |
| 1 (HS) | **1,049** | **21.3** |  | 433 | 24.0 | 457 | 18.9 | 107 | 23.8 | 49 | 21.2 |  |
| 2 (HS) | **2,090** | **42.6** |  | 814 | 45.1 | 988 | 40.9 | 190 | 42.3 | 98 | 42.4 |  |
| 3 (HS) | **764** | **15.6** |  | 244 | 13.5 | 423 | 17.5 | 70 | 15.6 | 27 | 11.7 |  |
| 4 or more (HS) | **916** | **18.6** |  | 295 | 16.3 | 499 | 20.7 | 75 | 16.6 | 47 | 20.3 |  |
| **Children in household** |  |  |  |  |  |  |  |  |  |  |  | <0.001 |
| Yes | **1,027** | **20.9** |  | 322 | 18.1 | 590 | 24.5 | 76 | 17.0 | 39 | 16.9 |  |
| No | **3,746** | **76.4** |  | 1,453 | 80.5 | 1,752 | 72.5 | 362 | 80.6 | 179 | 77.5 |  |
| **Adults 65 or over in household** |  |  |  |  |  |  |  |  |  |  |  | <0.001 |
| Yes | **1,042** | **21.3** |  | 435 | 24.1 | 476 | 19.7 | 86 | 18.9 | 45 | 19.5 |  |
| No | **3,792** | **77.4** |  | 1,353 | 74.9 | 1,906 | 78.9 | 356 | 79.3 | 177 | 76.6 |  |
| **Private health insurance** |  |  |  |  |  |  |  |  |  |  |  | 0.252 |
| No | **4,200** | **85.7** |  | 1,556 | 86.2 | 2,071 | 85.7 | 373 | 83.1 | 200 | 86.6 |  |
| Yes | **616** | **12.6** |  | 229 | 12.7 | 297 | 12.3 | 68 | 15.1 | 22 | 9.5 |  |
| **Voting preference** |  |  |  |  |  |  |  |  |  |  |  | <0.001 |
| Conservative | **1,162** | **23.7** |  | 528 | 29.2 | 455 | 18.8 | 132 | 29.4 | 47 | 20.3 |  |
| Labour | **1,290** | **26.3** |  | 403 | 22.4 | 733 | 30.3 | 96 | 21.4 | 56 | 24.2 |  |
| Liberal Democrat | **241** | **4.9** |  | 92 | 5.1 | 118 | 4.9 | 24 | 5.3 | 7 | 3.0 |  |
| Scottish National Party (SNP) | **203** | **4.1** |  | 73 | 4.0 | 99 | 4.1 | 23 | 5.1 | 8 | 3.5 |  |
| Green Party | **346** | **7.1** |  | 125 | 6.9 | 175 | 7.2 | 25 | 5.6 | 21 | 9.1 |  |
| Other | **721** | **14.7** |  | 301 | 16.6 | 310 | 12.8 | 73 | 16.0 | 37 | 16.1 |  |
| I would not vote | **249** | **5.1** |  | 68 | 3.8 | 137 | 5.7 | 22 | 4.8 | 22 | 9.5 |  |
| **Own health in general** |  |  |  |  |  |  |  |  |  |  |  | 0.185 |
| Very good | **881** | **18.0** |  | 328 | 18.2 | 420 | 17.4 | 93 | 20.4 | 40 | 17.3 |  |
| Good | **2,195** | **44.8** |  | 770 | 42.7 | 1,116 | 46.2 | 203 | 45.2 | 106 | 45.9 |  |
| Fair | **1,355** | **27.6** |  | 542 | 30.0 | 647 | 26.8 | 109 | 24.3 | 57 | 24.7 |  |
| Bad / Very bad | **399** | **8.1** |  | 145 | 8.0 | 194 | 8.0 | 39 | 8.7 | 21 | 9.1 |  |
| **Satisfaction with NHS** |  |  |  |  |  |  |  |  |  |  |  | <0.001 |
| Very satisfied | **1,272** | **25.9** |  | 536 | 29.7 | 566 | 23.4 | 117 | 25.7 | 53 | 22.9 |  |
| Fairly satisfied | **2,476** | **50.5** |  | 884 | 48.9 | 1,272 | 52.6 | 211 | 47.0 | 109 | 47.2 |  |
| Neither satisfied nor dissatisfied | **602** | **12.3** |  | 209 | 11.6 | 291 | 12.0 | 64 | 14.0 | 39 | 16.9 |  |
| Fairly dissatisfied / Very dissatisfied | **506** | **10.3** |  | 165 | 9.1 | 267 | 11.0 | 54 | 12.0 | 20 | 8.6 |  |
| **Experience with terminal illness** |  |  |  |  |  |  |  |  |  |  |  | 0.187 |
| No | **2,880** | **58.8** |  | 1,039 | 57.5 | 1,455 | 60.2 | 251 | 55.9 | 137 | 59.3 |  |
| Yes | **2,020** | **41.2** |  | 767 | 42.5 | 961 | 39.8 | 198 | 44.1 | 94 | 40.7 |  |
| **Influence on views** |  |  |  |  |  |  |  |  |  |  |  |  |
| Upbringing | **868** | **17.7** |  | 323 | 31.4 | 448 | 31.1 | 67 | 27.9 | 30 | 25.9 | 0.500 |
| Family | **1,098** | **22.4** |  | 341 | 33.0 | 617 | 39.8 | 75 | 34.2 | 65 | 43.6 | 0.004 |
| Education | **500** | **10.2** |  | 215 | 30.0 | 191 | 23.5 | 62 | 31.3 | 32 | 36.4 | 0.036 |
| Someone close to you having terminal illness | **1,105** | **22.5** |  | 434 | 52.7 | 533 | 48.0 | 103 | 52.6 | 35 | 38.9 | 0.204 |
| Work/professional life | **292** | **6.0** |  | 124 | 35.5 | 117 | 30.7 | 38 | 35.2 | 13 | 31.0 | 0.664 |
| Other ** | **1,038** | **21.7** |  | 369 | 35.5 | 509 | 49.0 | 212 | 20.4 | 56 | 5.4 |  |

* %’s are those within the viewpoint e.g. 51.5% of those on viewpoint 1 don’t belong to a religion

** Included ‘Media’, ‘Religious beliefs’, ‘Political orientation’, ‘individual events’ and ‘Clubs/societies’.

**Table A2: Descriptive statistics by statement**

| Statement Number (from initial Q study) |  | **Mean (1-7 scale)** | **Median** | **SD** |
| --- | --- | --- | --- | --- |
| 3 | Treatments should be directed towards people who have a greater chance of survival. | 5.06 | 5.00 | 1.66 |
| 5. | At the end of their life, patients should be cared for at home with a better quality of life rather than have aggressive and expensive treatments that will only extend life for a short period of time. | 5.47 | 6.00 | 1.49 |
| 26. | It is wrong to raise hopes and expectations by making a special case for treatments that will only extend life by a short time. | 4.77 | 5.00 | 1.79 |
| 38. | The health system should be about getting the greatest benefit overall for the population. | 5.77 | 6.00 | 1.39 |
| 2. | We should support an individual patient's choice for treatments that give short life extensions. | 3.21 | 3.00 | 1.72 |
| 13. | I would place more value on end of life treatments than many medical treatments for non-terminal conditions. | 4.52 | 4.00 | 1.65 |
|  | |  |  |  |
|  | **Viewpoint 2** |  |  |  |
| 17. | If a life extending treatment for terminally ill patients is expensive, but the only treatment available, it should still be provided. | 4.58 | 5.00 | 1.52 |
| 20. | We all have the right to life. | 5.90 | 7.00 | 1.52 |
| 27. | To extend life in a way that is beneficial to the patient is morally the right thing to do. | 5.47 | 6.00 | 1.49 |
| 37. | All human life is precious. | 5.94 | 7.00 | 1.54 |
| 1. | It is not worthwhile devoting more and more NHS money to someone who is going to die soon anyway. | 3.99 | 4.00 | 1.94 |
| 33. | End of life drugs are not a cure, they are life-prolonging. There is no point in delaying the inevitable for a short time. | 3.87 | 4.00 | 1.93 |
|  | |  |  |  |
|  | **Viewpoint 3** |  |  |  |
| 25. | We should spend proportionately more on patients when we feel those patients have not had their fair innings - in terms of the length of their life or the quality of that life. | - | - | - |
| 31. | Treatments that are very costly in relation to their health benefits should be withheld. | 3.79 | 4.00 | 1.80 |
| 34. | Patients at the end of life will grasp any slightest hope but that is not a good reason for the NHS to provide costly treatments that may extend life by a short time. | 4.50 | 5.00 | 1.87 |
| 41. | I wouldn’t want my life to be extended just for the sake of it - just keeping breathing is not life. | 5.76 | 7.00 | 1.68 |
| 23. | A year of life is of equal value for everyone. | 3.25 | 3.00 | 2.03 |
| 24. | You can't put a price on life. | 2.39 | 2.00 | 1.69 |
